# A centuries-long delay between a paleo-ice-shelf collapse and grounding-line retreat in the Whales Deep Basin, eastern Ross Sea, Antarctica

**DOI:** 10.1038/s41598-018-29911-8

**Published:** 2018-08-17

**Authors:** Philip J. Bart, Matthew DeCesare, Brad E. Rosenheim, Wojceich Majewski, Austin McGlannan

**Affiliations:** 1 Louisiana State University, Department of Geology and Geophysics, Baton Rouge, 70803 USA; 2 Auburn University, Department of Geosciences, Auburn, 36849 USA; 3 University of South Florida, College of Marine Sciences, St. Petersburg, 33701 USA; 4 Institute of Paleobiology, Polish Academy of Sciences, Warszawa, 00-818 Warszawa, Poland; 5 The University of Oklahoma, School of Geology and Geophysics, Norman, 73019 USA

## Abstract

Recent thinning and loss of Antarctic ice shelves has been followed by near synchronous acceleration of ice flow that may eventually lead to sustained deflation and significant contraction in the extent of grounded and floating ice. Here, we present radiocarbon dates from foraminifera that constrain the time elapsed between a previously described paleo-ice-shelf collapse and the subsequent major grounding-line retreat in the Whales Deep Basin (WDB) of eastern Ross Sea. The dates indicate that West Antarctic Ice Sheet (WAIS) grounding-line retreat from the continental shelf edge was underway prior to 14.7 ± 0.4 cal kyr BP. A paleo-ice-shelf collapse occurred at 12.3 ± 0.2 cal kyr BP. The grounding position was maintained on the outer-continental shelf until at least 11.5 ± 0.3 cal kyr BP before experiencing a 200-km retreat. Given the age uncertainties, the major grounding-line retreat lagged ice-shelf collapse by at least two centuries and by as much as fourteen centuries. In the WDB, the centuries-long delay in the retreat of grounded ice was partly due to rapid aggradational stacking of an unusually large volume of grounding-zone-wedge sediment as ice-stream discharge accelerated following ice-shelf collapse. This new deglacial reconstruction shows that ongoing changes to ice shelves may trigger complex dynamics whose consequences are realized only after a significant lag.

## Introduction

**Figure 1:**
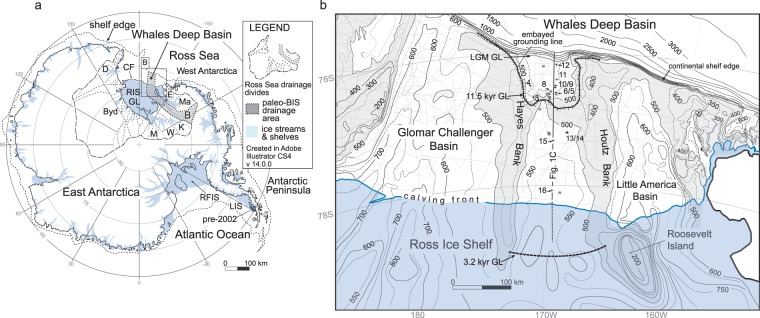
(**A**) Map of Antarctica showing ice streams and downstream (floating) ice shelves. The dashed lines in the continental interiors demarcate drainage areas of the East and West Antarctic Ice Sheets that converge into Ross Sea. The gray shade shows the paleo-Bindschadler Ice Stream drainage area during the LGM. B = Bindschadler Ice Stream; D = David Glacier; Byd = Byrd Glacier; M = Mercer Ice Stream; W = Whillans Ice Stream; K = Kamb Ice Stream; Ma = MacAyeal Ice Stream; E = Echelmeyer Ice Stream; CF = calving front; RIS = Ross Ice Shelf; GL = grounding line; RFIS = Ronne-Filchner Ice Shelf; LIS = Larsen Ice Shelf with the darker blue area showing the extent of the Larsen Ice Shelf that collapsed in 2002. The modern-day shelf edge position is shown as a dashed line. (**B**) Bathymetry of the eastern Ross Sea continental shelf. The paleo-BIS was confined to the WDB between the Hayes and Houtz Banks. The light gray rectilinear lines are the locations of seismic data. The squares are core acquired by Mosola and Anderson^24^ and the crosses are those described by McGlannan *et al*.^18^.

The floating ice shelves that fringe Antarctic Ice Sheets (AISs) are an important part of the cryosphere because they slow the offshore flow of grounded ice. Hence, the presence/absence of an ice shelf greatly influences the balance between ice-volume accumulation and ablation^1^. In the present interglacial climate, ice shelves are melting from their upper^2,3^ and lower surfaces^4–7^. The current rapid rate of thinning reduces the buttressing effect of ice shelves^8^. The collapse of the Larsen Ice Shelf B on the Antarctic Peninsula in 2002^9^ (Fig. 1a) triggered a near instantaneous accelerated flow of the inland ice streams^10^. The associated ice-stream deflation is of concern because it may eventually lead to major grounding-line retreat^8,11^, especially in those areas where marine-based ice overlies a foredeepened seafloor^12^. There is, however, a dearth of paleo-data that constrains the geologic timeframes over which dynamic thinning and deflation trigger significant and sustained grounding-line retreat after ice-shelf collapse^13,14^. Grounding-line retreat is crucial to assessing whether ongoing AIS changes significantly contribute to global sea level rise. This leads to the following question: how much time elapsed between paleo-ice-shelf collapse and significant dynamic response in the form of paleo-grounding-line retreat? Several previous reconstructions have shown that paleo-ice shelves have experienced abrupt collapse^15–18^. The interplay between paleo-ice-shelf collapse and grounding-line retreat has not previously been constrained due to general paucity of reliable dates from the sedimentologies and geomorphologic features that record calving-front and grounding-line oscillations. A paleo-perspective of changing ice dynamics in response to ice-shelf collapse is crucial to understanding the potential sensitivity of ice stream discharge to ice shelf thinning^19^ amidst recent observations that grounding lines are retreating throughout Antarctica^20^.

Here, we present radiocarbon dates of *in situ* foraminifera from sub-ice-shelf and grounding-line-proximal sediments to constrain the time elapsed between a paleo-ice-shelf breakup and the subsequent major grounding-line retreat in the WDB of eastern Ross Sea. The development of this retreat chronology specifically relied on a detailed stratigraphic framework that was presented in two recent studies^18,21^.

## Background

### Reconstructing ice-sheet retreat from the geology of continental shelf trough basins

During the Last Glacial Maximum (LGM), i.e., from 24,000 to 19,000 years ago, mass balance had shifted to positive and the extent of ice flow from East and West Antarctica expanded towards the Antarctic continental shelf edge (Fig. 1a)^21–26^. Broad and wide bathymetric troughs and banks on the outer continental shelves demonstrate that several fast-flowing erosive ice streams formerly drained the AISs (Fig. 1a,b). In Ross Sea, ice flow converged into five large ice-streams that reached the outer continental shelf (Fig. 1a). The subsequent retreat of grounded and floating ice is well recorded in the sedimentology and geomorphology of paleo-ice-stream trough basins that were partly backfilled with grounding zone wedges (GZWs) and post-glacial sediment successions^21,25–28^. The former locations of the ice-sheet grounding line are best reconstructed from the morphological boundary between a gently, landward-dipping GZW topset (the surface to which the ice stream was grounded) and a steeply seaward-dipping GZW foreset (the basinward-dipping marine depositional surface). In other words, GZWs are essentially subaqueous moraines deposited at the marine termination of the ice stream. Identifying and mapping GZW morphology on seismic profiles and bathymetric data is crucial to accurately reconstruct AIS retreat because these features represent the former locations occupied by grounded ice and thus provide unequivocal evidence that ice streams became stationary, or momentarily re-advanced, during their overall retreat. Early deglacial reconstructions were routinely based on reconnaissance level surveys of the major trough basins^24^. The physical reconstructions have continually been improved as more marine data have been acquired. These improved stratigraphic frameworks are important because they highlight the locations from which a chronology of ice-sheet and ice-shelf retreat from the outer continental shelves can best be obtained.

### WAIS retreat from the WDB outer and middle continental shelf

**Figure 2:**
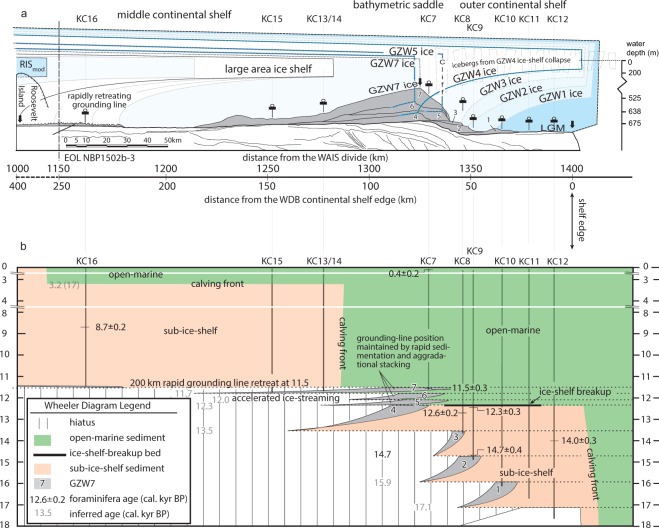
(**A**) Line drawing interpretation of regional dip-oriented seismic line NBP1502B_3 with projected core stations shown as vertical lines. The transect location is shown with a solid bold line in B. At the southern end, we show our projection of stratal relationships below the Ross Ice Shelf. The gray shaded units labeled 1–7 are GZWs^21^ with the successive ice sheet and ice shelf positions occupied through time^18^. The multiple grounding line and ice shelf positions shown after GZW7 represent rapid retreat of the grounding line to Roosevelt Island. RIS_mod_ = the modern position of the Ross Ice Shelf. (**B**) Wheeler Diagram corresponding to line drawing interpretation of NBP1502B_3 (shown in **A**). The diagram shows distance along the earth’s surface on the horizontal axis. Unlike the seismic section, the vertical axis of a Wheeler Diagram represents geologic time. In other words, any horizontal line through the diagram is an isochron representing lateral changes in paleoenvironmental setting. The different colored zones on the diagram show both the horizontal and vertical changes in sedimentary environments through time as constrained by seismic stratigraphy and seafloor geomorphology as well as sediment facies and radiocarbon ages. The core stations used to construct the sedimentary facies shown on the diagram are posted on the top of the Wheeler Diagram. The key foraminiferal radiocarbon dates from this study are posted adjacent to the black vertical lines, whose lengths represent the sediment penetration at the various core stations. The gray numbers are inferred ages. The 3.2 kyr date is from Conway *et al*.^47^.

During the austral summer of 2015, the distribution of sea ice was low and sea state was unusually mild in eastern Ross Sea. Those favorable conditions allowed acquisition of a large-area (2,500 km^2^) multibeam swath bathymetry survey, three regional dip-oriented seismic reflection profiles from the center of the WDB and a transect of strategically-located core during expedition *NBP1502B* (Figs 1b, 2a). The WDB was formerly occupied by an expanded Bindschadler Ice Stream, which received ice flow from the central part of the WAIS. Retreat of the Bindschadler Ice Stream from WDB has been recently well-constrained using both detailed analysis of seafloor morphology and core sedimentology. Multibeam swath bathymetric surveys coupled with subsurface stratigraphic imaging^21^ revealed that the paleo-Bindshadler ice stream (paleo-BIS) had advanced to the continental shelf edge as evidence by megascale glacial lineations (MSGLs). A backstepping succession of seven GZWs that partly bury the earlier-formed MSGLs marks the initial retreat of the groundling line from the continental shelf edge. This overlapping stack (GZW1 through 7) indicates that the grounding line paused multiple times as the ice stream vacated the WDB (Fig. 2a)^21^; four of the seven GZWs are partly exposed on the outer continental shelf of the WDB^21^. Grounding zone wedges 4 through 7 exhibit an overall aggradational stacking pattern that is manifest as the large bathymetric saddle between the Hayes and Houtz Banks located approximately 70 km from the continental shelf edge (Fig. 1b). In contrast, the lack of GZWs on the middle continental shelf, i.e. south of the bathymetric saddle, suggests that after the deposition of GZW7, the grounding line abruptly shifted 200 km towards Roosevelt Island (Figs 1b, 2a). Only a series of small morainal ridges that mantle the topset of GZW7 on the middle continental shelf record the major retreat.

The favorable conditions during the *NBP1502B* expedition allowed strategic coring with respect to the exposed GZWs to reconstruct the patterns with which the grounding line and the calving front oscillated between groundings^18^. Most of the cores penetrated to massive diamict that was deposited either in a subglacial or grounding-line-proximal setting as the grounding line was retreating across the outer continental shelf. The upcore sediment transitions from diamict to glacial marine sediments (representative of sub-ice-shelf and open-marine environments) record the time-transgressive retreat of both grounded and floating ice. The correlation of the geophysical and geological data permitted a much more detailed reconstruction of WAIS retreat^18^ (Fig. 2a) than was possible with previously available reconnaissance-level data from the WDB^24^. Most importantly, although the grounding line paused seven times during retreat, no major back and forth oscillations of either grounded or floating ice occurred between these groundings^18^. The paleo-ice shelf that formerly covered the outer continental shelf collapsed during the deposition of GZW4^18^ (Fig. 2a). Despite ice-shelf collapse, the regional stratigraphic framework requires that the grounding line remained on the outer continental shelf to deposit GZWs 5 through 7 in an overall aggradational stacking pattern^18^. Grounded ice then abruptly retreated an additional 200 km to Roosevelt Island (on the inner continental shelf) and a large ice shelf reformed over the middle continental shelf (Fig. 2a)^18^. The Ross-Ice-Shelf calving front eventually moved south to its modern position. The combination of detailed surface and subsurface mapping of the WDB with strategic coring allowed by anomalously good sea state and sea ice conditions allows for tremendous insight into the former grounding locations as well as the precise stratigraphic boundaries in sediment cores that record the key transitions in paleo-environmental conditions that record calving-front and grounding-line oscillations.

### Foraminiferal record from the WDB

**Figure 3:**
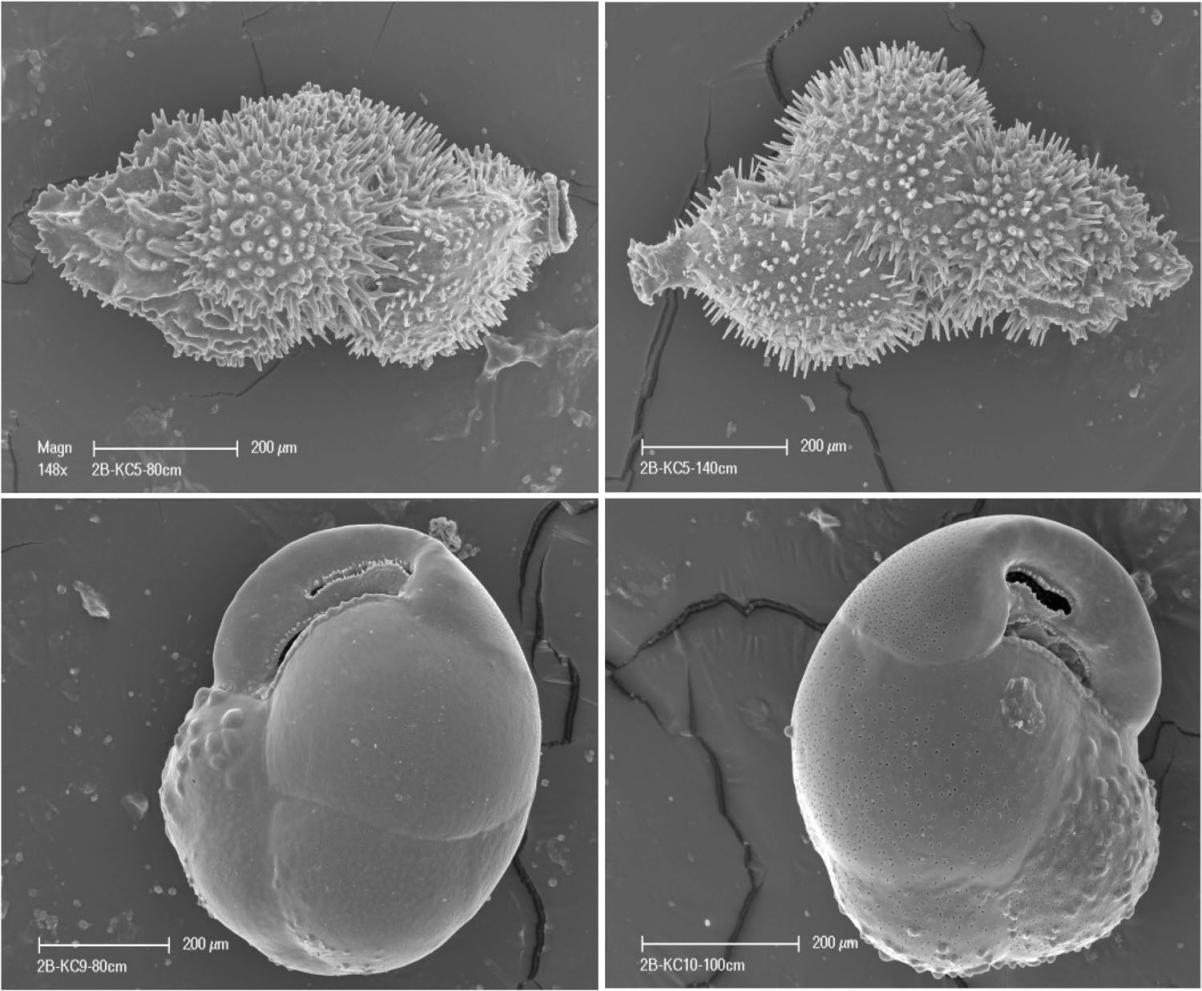
SEM images of pristine preservation among calcareous foraminifera from cores recovered during expedition *NBP1502B*; *Trifarina earlandi* specimens are shown in the upper row and *Globocassidulina biora* specimens are shown in the lower row.

A recent study by Majewski *et al*.^22^ found that ice-proximal sediments in the WDB contain relatively abundant *in situ* foraminifera. Benthic and/or planktonic foraminifera were found in all cores, however, the abundance and species composition differed between and throughout each core. In general, the upper parts of the cores down to 10 to 20 cm below the sediment/water interface represented open-marine conditions and contained agglutinated foraminifera with abundances and diversities that decreased with depth. Agglutinated foraminifera were dominated by *Miliammina arenacea* associated with *Paratrochammina*, *Reophax*, *Spiroplectammina* and *Labrospira*^22^. Below 10 to 20 cm core depth, calcareous foraminifera were present at most locations except from cores on the middle continental shelf (i.e., KC14, KC15, and KC16), which were almost barren of calcareous foraminifera. In contrast, in all underlying sediments on the outer continental shelf, the total foraminiferal abundances were variable but strongly dominated by calcareous foraminifera, reaching in some intervals up to 40 specimens per gram of dry sediment. Taxonomic composition, although limited to less than twenty-five species in total, was variable^22^. The calcareous assemblage was always dominated by *Globocassidulina* and/or *Trifarina*, accompanied by *Cibicides*, *Ehrenbergina*, *Astrononion*, *Epistominella*, and other benthic genera, in addition to the planktonic *Neogloboquadrina pachyderma* sinistral. Preservation of calcareous foraminifera was variable but mostly ranged from fair to pristine. Some broken or etched tests were encountered but well-preserved, complete, transparent tests with no sediment infill were dominant in the most fossiliferous intervals. Some examples of pristine preservation with intact fragile spines, no signs of wall dissolution, and no secondary filling of very fine pores were present (Fig. 3).

## Methods

The detailed understanding of regional geomorphology^21^, sedimentology^18^ and foraminiferal presence^22^ outlined above is an essential framework to our study of retreat chronology in the WDB. With respect to our objective of constraining the time elapsed between paleo-ice-shelf collapse and major grounding-line retreat in the WDB, the stratigraphic framework shows that there are two key sedimentary facies transitions that have to be dated. The first transition is the ice-shelf collapse during GZW4 that is recorded in core on the outer continental shelf by the sedimentary change from the sub-ice-shelf to ice-shelf-breakup units. The second transition is the subsequent major grounding line retreat at the end of GZW7 that is recorded in core from the GZW7 foreset by the sedimentary change from diamict to open-marine diatom ooze. Our methodology focused on radiocarbon dating of well-preserved foraminifera isolated from near these two sedimentary boundaries. In instances where foraminifera were not sufficiently abundant for ^14^C analysis near the facies boundary, the closest interval below the sedimentary facies boundary was used.

### Foraminiferal selection

Sediment samples (150 cm^3^ volume) were collected from all kasten cores (KC) at intervals of 5 to 10 cm for foraminiferal analyses directly after onboard visual core description. Care was taken to not include abrupt facies transitions in a single sample. The jumbo piston cores (JPCs) were cut and sampled using a pair of 50 cm^3^ sediment plugs in July 2015 after their arrival at the Antarctic Marine Geology Research Facility in Tallahassee, Florida. Sediment samples were collected every 10-cm using 2 cm thick core intervals. Samples directly below and above each facies transition were processed using the methods outlined below. These samples did not always yield enough biogenic carbonate in pristine condition for radiocarbon analysis. In these instances, preliminary foraminiferal abundances from Majewski *et al*.^22^ were used as a reference for sample selection.

Sediment was washed through a 63 μm sieve to remove silt and clay fractions and dried at ~50 °C. Sediment was then dry-sieved using 149, 250 and 425 μm fractions before collecting foraminifera. Tests were picked under a binocular light microscope and placed into microslides for archiving. Specimens were identified to the species level. Only unbroken tests that did not exhibit any signs of discoloration breakage were isolated using a binocular light microscope and used in further analysis. A minimum of 0.6 mg of calcite was measured for radiocarbon analysis.

### Radiocarbon analyses

Foraminiferal samples were sent to the University of South Florida College of Marine Science to be converted to CO_2,_ along with modern and ^14^C-free carbonate reference materials, by reaction with phosphoric acid (H_3_PO_4_) in vacuo. Glass ampoules of CO_2_ gas were sent to Woods Hole Oceanographic Institute to be analyzed for ^14^C on the National Ocean Sciences Accelerator Mass Spectrometer. One sample of *G. biora* was split into two equal masses and replicate ages were within 50 ^14^C yr of one another. These data were calibrated to calendar year BP using MARINE13 calibration curve in CALIB 7.1^29,30^ with a marine reservoir effect of 1,300 ± 100 yr (differing by 900 yr from the global average reservoir age of 400 yr) established for Antarctic marine carbonates^31^. All foraminiferal radiocarbon ages are in cal kyr BP as the median age ± the age range representing the 95% confidence interval as per radiocarbon reporting conventions^32^. All data generated or analyzed during this study are included in this published article.

## Results

### Radiocarbon ages of foraminifera in the WDB

Foraminifera dated for this study came from intervals that showed the most abundant calcareous foraminifera^22^. Where possible, we used monospecific samples comprised of benthic *G. biora* and *T. earlandi* (and in one instance planktonic *N. pachyderma*) that were the most numerous and fairly massive (Table 1). The good to pristine preservation of these foraminifera, along with the lack of clearly reworked specimens, that could be identified either by different coloration, sediment filling^33^ and/or presence of extinct species^34^, indicates that practically all specimens could be considered as being *in situ*^22,35^. Rare specimens showing signs of etching, found especially among *Cibicides* or foraminiferal specimens in cores from the middle continental shelf, although most likely also being *in situ*, were not used for radiocarbon analysis.

**Table 1:** Radiocarbon ages for foraminifera.

Sample #	Core ID		Interval (cm)	Lithology	Species	14 C yr BP	Age Err ±	Fm error	δ13C	Calibratd yr BP	±age range	range −	range +
*OS-126095		KC12	38–42	SIS	mixed benthic	12,950	65	0.001700	−0.11	13,504	221	13,283	13,742
OS-122390		KC12	53–58	SIS	*T. earlandi*	13,405	120	0.002833	−0.58	13,990	256	13,734	14,246
*OS-126094		KC12	97–102	SIS	mixed benthic	13,200	75	0.001800	0.09	13,763	252	13,511	14,015
OS-122389		KC12	215–220	diamict	*G. biora*	44,837	4,200	0.002265	−0.29	46,651	512	46,139	47,163
OS-122357		KC11	80–85 A	SIS	*G. biora*	12,597	70	0.001808	−0.1	13,177	223	12,954	13,400
OS-122358		KC11	80–85B	SIS	*G. biora*	12,644	70	0.001809	−0.11	13,221	217	13,004	13,438
OS-131664		KC11	80–85	SIS	*N. pachyderma*	12,550	35	0.00090	0	13,130	228	12,880	13,335
OS-122363		KC11	180–185	SIS	*G. biora*	13,201	80	0.001920	−0.56	13,765	252	13,513	14,017
OS-122364		KC10	100–105	SIS	*G. biora*	13,162	80	0.001915	0.36	13,720	245	13,475	13,965
OS-122394		KC09	30–35	SIS	*T. earlandi*	11,702	150	0.004432	−0.56	12,268	319	11,949	12,587
OS-122368		JPC09	235–237	SIS	*G. biora*	13,797	85	0.001925	0.31	14,683	449	14,234	15,132
OS-122391		KC08	40–45	SIS	*T. earlandi*	12,010	110	0.003136	−0.51	12,630	202	12,428	12,832
OS-122388		JPC07	235–237	diamict	*G. biora*	11,664	85	0.002532	0.54	12,214	346	11,868	12,560
OS-122360		KC06	146–148	diamict	*G. biora*	11,818	75	0.002115	0.04	12,421	281	12,140	12,702
OS-122385		KC05	64–69	diamict	*G. biora*	11,268	90	0.002725	0.65	11,472	341	11,131	11,813
OS-122359		JPC05	280–282	diamict	*G. biora*	11,432	70	0.002116	0.73	11,740	416	11,325	12,156
OS-122383		KC03	20–25	OM	*N. pachyderma*	1,618	35	0.003302	0.68	357	197	160	554
OS-131665		KC16	200–204	SIS	mixed benthic	8,660	30	0.00130	0.47	8,223	201	8,001	8,403

Sample numbers are from NOSAMS. The asterisks symbol denotes samples that are mixed benthic species. 14 C yr BP is uncalibrated ages. Calibrated yr BP is the median age results from Calib 7.1 software with ± age range being the 95% confidence interval. SIS = sub-ice shelf; OM = open marine.

We obtained a total of eighteen foraminiferal radiocarbon ages (Table 1). Seventeen of the new radiocarbon dates presented here were generated from benthic foraminifera. The calibrated age uncertainties are low with fifteen of the eighteen dates having uncertainties ranging from 197 to 346 years (analytical uncertainties ranged between 30–150 ^14^C y). The largest uncertainty, from the smallest sample mass, was 512 years. Eleven dates were obtained from sub-ice-shelf sediments that accumulated on the outer continental shelf prior to the ice-shelf collapse. Only two of the median calendar ages from the sub-ice-shelf unit on the outer continental shelf are out of stratigraphic order. Given the possible range of calibrated ages represented by the median dates, however, the ages are considered to be in good stratigraphic order. The oldest date from the base of sub-ice-shelf sediments (in KC9) is 14.7 ± 0.4 cal kyr BP. The youngest date from the top of the sub-ice-shelf sediments (in KC8) is 12.3 ± 0.3 cal kyr BP. To better constrain the range of timing for sub-ice-shelf sedimentation on the outer continental shelf, two samples from KC12, comprised of mixed benthic species *G. biora* and *T. earlandi*, were radiocarbon dated. These ages are 13.5 ± 0.2 cal kyr BP at 38 cm depth and 13.8 ± 0.3 cal kyr BP at 97 cm depth. These data essentially overlap with the monospecific age 14.0 ± 0.3 at 53 cm and therefore also represent viable radiocarbon ages for sub-ice-shelf sedimentation on the outer continental shelf prior to ice-shelf collapse. No radiocarbon dates were obtained for the ice-shelf-breakup unit. Only one radiocarbon date, 0.4 ± 0.2 cal kyr BP, was obtained from the upper part of the open-marine sediment on the outer continental shelf at KC03.

Four radiocarbon dates were obtained from foraminifera (exclusively *G. biora*) that were isolated from diamict sediments that accumulated on GZW7 foreset on the outer continental shelf. The ages come from three core sites that are separated by 50 km. In the one core with multiple dates, i.e., KC05, the calendar ages are in stratigraphic order. The age uncertainties range from 281 to 416 years. The calendar ages range from 12.4 ± 0.3 cal kyr BP to 11.5 ± 0.3 cal kyr BP. No radiocarbon dates were obtained for open-marine sediments that overly the GZW7 diamict because those sediments contained only a few calcareous foraminifera. The new radiocarbon dates (Table 1) are shown with respect to the lateral sedimentary facies changes on a Wheeler Diagram (Fig. 2b).

## Discussion

A total of eleven radiocarbon dates from six core sites (KC12, −11, −10, −9, −8 and −6; see Table 1) constrain the timing of sub-ice-shelf sedimentation on the WDB outer continental shelf prior to the paleo-ice-shelf collapse. The oldest radiocarbon date from sub-ice-shelf sediment, 14.7 ± 0.4 cal kyr BP, comes from core station KC09 (Fig. 2a,b). Its stratigraphic location atop diamict deposited on the GZW2 foreset indicates that a third grounding-line shift away from the continental shelf edge (i.e., the establishment of a grounding line associated with GZW3 with its adjacent sub-ice-shelf environment) had occurred by 14.7 ± 0.4 cal kyr BP, (Fig. 2a,b; Table 1). Likewise, the geologic principles of stratigraphic superposition and facies relationships (in particular, the existence of sub-ice-shelf sediments above the GZW3 foreset at core station JPC/KC08, Fig. 2a,b) require that a small ice shelf continuously covered the outer continental shelf by the time that the grounding line had experienced a fourth shift away from the continental shelf edge (as recorded by shift from GZW3 to GZW4)^18^. The foraminiferal radiocarbon dates indicate that the ice shelf remained in existence until at least 12.3 ± 0.2 cal kyr BP (Fig. 2b) but its collapse occurred shortly thereafter and is recorded by an ice-shelf-breakup event bed (Fig. 2a,b)^18^. The youngest date from sub-ice-shelf sediments, 12.3 ± 0.2 cal kyr BP, is from immediately below the ice-shelf-breakup unit in KC8. The area of the ice shelf that collapsed was likely similar to that of the Larsen B Ice Shelf that collapsed in 2002^36,37^.

Based on the regional stratigraphic framework, the loss of ice-shelf buttressing was coeval with the end of GZW4^18^ (Fig. 2b). After ice-shelf collapse, a grounding-line ice cliff would have existed at the grounding line (Fig. 2b). To sustain such a configuration, a rapid increase in ice stream flow^38,39^ and an increase in sediment flux would have accompanied it. In the WDB, this is recorded by a significant change from backstepping (GZW1 through −4) to progradational/aggradational (GZW5 through −7, see Fig. 2a). Our radiocarbon ages (Table 1) provide strong support that the significantly larger volume GZWs (GZW5 through −7, see Fig. 2a,b) were the result of rapid deposition that would have been associated with increased ice stream flow. In the WDB, the grounding-line position on the outer continental shelf was apparently maintained by rapid subglacial and grounding-zone sedimentation^40^ as represented by the aggradational stacking of GZW4 through −7 which filled the accommodation created by ice-stream deflation from accelerated ice-stream flow^21,41^.

A total of five radiocarbon dates from four core sites (KC3, −5, −6 and −7; see Table 1) constrains the timing of diamict deposition on the GZW7 foreset. Our estimated date for the major grounding line retreat that followed paleo-ice-shelf collapse is based on the youngest age of benthic foraminifera from GZW7 diamict, 11.5 ± 0.3 cal kyr BP. This date is taken from immediately below the sedimentologic boundary that marks the abrupt transition to an open-marine setting in KC7. Bart *et al*.^35^ showed that calcareous foraminifera from GZWs can be used for radiocarbon dating of marine diamicts from the eastern Ross Sea if they are selected with considerable care.

Taken at face value, the median radiocarbon ages for paleo-ice-shelf collapse and major grounding-line retreat (i.e., 12.3 and 11.5 cal kyr BP, respectively) suggest that the grounding-line position on the WDB outer continental shelf was maintained for eight centuries after the ice shelf collapse. Fully considering the calibration-induced age ranges, the grounding-line position persisted for a minimum of 200 years and a maximum of 1400 years after ice shelf breakup. In a geologic context, even our maximum estimate could be considered an instantaneous response. As concerns human-relevant time scales, an important point here is that the ongoing changes to ice shelves may trigger complex dynamics whose consequences are realized only after a significant lag. The land-based glaciers that were formerly fronted by the Larsen Ice Shelf B experienced immediate acceleration following the ice-shelf collapse in 2002 however, there has of yet been no significant retreat in the grounding line. In the case of the WDB, a centuries-long lag necessitates sediment aggradation from a high sediment flux. Sediment flux to the grounding line depends on several factors including the ice-sheet drainage area^42^ as well as the substrate erodibility and the presence/absence of melt water^43,44^. In the case of the WDB, easily erodible unconsolidated Plio-Pleistocene strata underlie the WDB^45^ and hence the paleo-BIS sediment flux was sufficiently high to deposit an unusually large-volume compound GZW^41^ that helped maintain a relatively stationary grounding line.

In the WDB, the subsequent 200-km retreat of the grounded line toward Roosevelt Island constituted an abrupt additional 15% contraction in the extent of grounded ice. A large-area ice shelf reformed over the middle continental shelf (Fig. 2a,b; see the upcore transitions from subglacial till to sub-ice-shelf sediments at KC13 thru KC16). A radiocarbon date from the middle continental shelf (at KC16) indicates that this second ice shelf was still in existence by 8.6 ± 0.2 cal kyr BP (Fig. 2a,b). Compound specific radiocarbon dates suggest that the calving front had migrated south of the middle continental shelf before 5 kyr BP^46^ but there is no sedimentologic evidence that a second catastrophic breakup of the reformed ice shelf that covered the middle continental shelf. Grounded ice eventually retreated past Roosevelt Island at 3.2 kyr BP^47^.

Thus far, all consideration of uncertainty of the timing of paleo-BIS deglaciation has incorporated analytical uncertainty (relatively small) and calibration uncertainty (larger). Certainly, the sub-ice-shelf melting, if comparable to today, could possibly have been driven at least in part by a water mass with an older age (greater reservoir age). Deep-sea stylasterid hydrocorals record radiocarbon age differences during Circumpolar Deep Water (CDW) incursion of about 100–200 years (King *et al*., in review). This is less than, but a significant portion of, the minimum amount of lag between ice shelf breakup and grounding line retreat that our foraminifera dates constrain. Given that the reservoir age of warmer water masses (e.g., today’s CDW) is related to far field processes that create it in the north Atlantic as well as local processes that pull it onto the continental shelf, it is difficult to postulate the potential for a large difference in reservoir age during deglaciation compared to what is observed today. Indeed, there is evidence of a slowdown of North Atlantic Deep Water formation, which would potentially have increased reservoir ages during the LGM^48^. However, the overall properties of Southern Ocean water masses also involve more local processes related to the Antarctic Circumpolar Current and shelf-slope exchange. These are less well constrained, limiting how much we can forecast differences in the reservoir age of subsurface, potentially warm water masses that could have driven the ice dynamics preserved in WDB sediments.

The only previous study of the WAIS retreat from the eastern Ross Sea outer continental shelf relied on bulk acid-insoluble organic matter isolated from deglacial sediment^24^. Their core stations in the WDB are not ideally located and hence they are not useful for our study. More importantly, they considered their radiocarbon dates suspect because the bulk organic matter yielded very old ages at the sediment-water interface. Apparently, older organic carbon is mixed with that generated in the water column contemporaneous with deglacial sedimentation, a prognosis that is typical in Antarctic margin sediments^49^. Our results confirm that a highly-detailed retreat chronology from *in situ* foraminifera can be obtained via synthesis of regional-scale geophysical surveys and targeted sediment coring. A combination of factors (collapse of the ice shelf that covered the outer continental shelf, accelerated flow and ice-stream deflation, as well as the aggradation of a foredeepened profile by the end of GZW7 deposition) all predisposed the paleo-BIS to its subsequent rapid major (200 km) retreat to Roosevelt Island at 11.5 ± 0.3 cal kyr BP (Fig. 2b). The negative mass balances associated with ice-shelf collapse, accelerated ice-stream discharge and eventual grounding-line shift would have delivered freshwater to the Southern Ocean with the potential to alter thermohaline circulation and other aspects of the global climate system^50^.

A review of the literature on Antarctic margins indicates that many ice streams experienced backstepping retreats of floating and grounded ice, but, given uncertainties in calibrations for different dating strategies, it is not yet possible to definitely say whether the WDB ice-shelf collapse and grounding-line shifts were synchronous with ice-sheet changes recorded elsewhere^25,51–55^. The WDB ice-shelf collapse may have been synchronous with an increase in iceberg rafted debris flux in Scotia Sea referred to as AID2 by Weber *et al*.^53^. Their composite records of ice-rafted debris from the Scotia Sea permits the possibility that either pulsed ice-shelf collapse, subsequent accelerated ice-stream discharge or abrupt grounding-line retreat may have ultimately caused the increased iceberg rafting fluxes^53^.

Calving-front and grounding-line shifts are significant measures of climate change. The geological perspective provided by these results are important to climate models that seek to predict dynamic grounding-line and calving response to either external and/or internal forcing^50,56,57^. The new detailed reconstruction from the WDB shows that a paleo ice-shelf collapse triggered complex dynamics whose consequences were not fully realized until after a lag of at least two centuries. Even in isolation, the ice volumes returned to the global ocean from the WDB in the time elapsed since the paleo-ice-shelf collapse at 12.3 ± 0.2 cal kyr BP and subsequent 200-km grounding-line retreat at 11.5 ± 0.3 cal kyr BP were relevant to coastal settings in terms of the resulting sea-level rise and coastal transgressions it would have produced. As concerns the potential for future ice-shelf collapse in the present-day interglacial, it is worth noting that many ice-stream systems are now grounded on the foredeepened inner continental shelves that is presumably underlain by either basement rock or other less erodible substrate. Hence, a transition to unbuttressed flow of those modern ice streams might be less able to maintain a grounding position by accelerated deposition of sediment within the grounding zone.
